# Cancer without borders: Policy frameworks for oncology care in humanitarian and conflict settings

**DOI:** 10.18632/oncotarget.28856

**Published:** 2026-03-31

**Authors:** Pragnesh Parmar, Gunvanti Rathod

**Affiliations:** ^1^Forensic Medicine and Toxicology, AIIMS, Bibinagar, Telangana, India; ^2^Pathology and Lab Medicine, AIIMS, Bibinagar, Telangana, India

**Keywords:** cancer care, humanitarian crisis, tele-oncology, global health policy, oncology triage

## Abstract

Cancer is an escalating yet neglected health crisis among refugees, migrants, and populations affected by conflict. Despite increasing global focus on non-communicable diseases (NCDs), oncology remains largely absent from humanitarian health agendas. This narrative review synthesizes evidence from peer-reviewed literature, humanitarian agency reports, and case studies from Gaza, Sudan, and Ukraine to examine the policy, ethical, and clinical dimensions of oncology care in crisis settings. Findings reveal systemic neglect of cancer services due to disrupted infrastructure, legal barriers, and fragmented policy frameworks. Vulnerable groups - women, children, and the elderly - experience the greatest inequities. Ethical dilemmas in triage, limited palliative care, and inadequate digital connectivity further hinder equitable access. Emerging solutions include bilateral treatment agreements, WHO-led humanitarian oncology corridors, and tele-oncology or mobile unit models that sustain care across borders. Addressing cancer in humanitarian contexts is not merely a technical challenge but a moral imperative. Integrating oncology into emergency response protocols and global health governance is essential to ensure continuity, dignity, and justice in care for displaced and conflict-affected populations.

## INTRODUCTION

### The borderless burden of cancer

The global burden of cancer has steadily risen over the past decades, with an estimated 20 million new cases and 10 million cancer-related deaths reported worldwide in 2022 alone, disproportionately affecting low- and middle-income countries (LMICs) [1]. While global oncology initiatives have increasingly focused on strengthening national cancer control plans, screening, and treatment infrastructures, there remains a critical blind spot in addressing the needs of forcibly displaced populations - including refugees, asylum seekers, internally displaced persons (IDPs), and undocumented migrants - particularly in humanitarian and conflict settings [2, 3].

Cancer, traditionally framed as a non-urgent, chronic condition, is often excluded from the priority list during humanitarian emergencies. Health systems overwhelmed by trauma care, infectious disease outbreaks, and basic survival needs often relegate oncology to a secondary or even invisible status [4]. Moreover, displaced individuals face a constellation of systemic barriers that impede timely diagnosis, treatment initiation, and continuity of care. These barriers include disrupted health records, cross-border legal complexities, lack of cancer registries, stigma, and logistical challenges such as transportation and affordability [5–7]. A recent analysis by the United Nations High Commissioner for Refugees (UNHCR) and WHO observed that non-communicable diseases (NCDs), including cancer, account for over 50% of morbidity in displaced populations, yet remain chronically underfunded in humanitarian response budgets [8].

In conflict-affected regions such as Gaza, where infrastructure collapses under siege; Sudan, where civil war dismantles public health systems; or Ukraine, where millions have been displaced across borders, the plight of oncology patients is often reduced to fragmented, reactive, and ethically fraught interventions [9–11]. Despite growing recognition of the need to integrate NCD care into emergency responses, oncology continues to suffer from a policy vacuum. Humanitarian health guidelines like the Sphere Handbook lack oncology-specific standards, and most emergency medical teams (EMTs) deployed in crises are not equipped to deliver chemotherapy, radiotherapy, or palliative cancer services [12].

This review aims to synthesize current knowledge, policy gaps, and operational challenges in providing cancer care to displaced populations, with a particular focus on humanitarian and conflict settings. It draws on cross-national case studies, reviews existing legal and ethical frameworks, and explores feasible models such as cross-border care agreements, tele-oncology, and triage systems adapted for oncology in crisis zones. The goal is to reframe cancer care not as a luxury, but as a humanitarian imperative - and to chart actionable pathways for global oncology equity in times of conflict and displacement.

## METHODOLOGY

This study followed a narrative review approach to synthesize existing knowledge on cancer care in humanitarian and conflict settings. Relevant literature published between 2010 and 2025 was identified through searches of PubMed, Scopus, and Google Scholar, using combinations of keywords such as *“cancer care,” “refugees,” “migrants,” “humanitarian crisis,” “conflict zones,” “non-communicable diseases,”* and *“oncology policy.”* Additional grey literature - including policy reports, technical briefs, and situation analyses - from WHO, UNHCR, Médecins Sans Frontières (MSF), NCD Alliance, and World Bank was also reviewed to capture field-level data and operational perspectives.

Inclusion criteria comprised:

1. Publications addressing oncology or cancer-related service delivery in displaced, migrant, or conflict-affected populations;
2. Studies or reports analyzing ethical, legal, or policy aspects of cancer care in humanitarian contexts;
3. Sources providing quantitative, qualitative, or mixed-methods data relevant to access, infrastructure, or health equity; and
4. Documents available in English with sufficient methodological transparency or institutional credibility.

Exclusion criteria included duplicate publications, commentaries lacking empirical or policy content, and non-English sources without reliable translation. Data were extracted and thematically synthesized into key domains: epidemiology and access barriers, ethical dilemmas, policy and legal frameworks, and innovative care models such as tele-oncology and mobile units.

### The policy vacuum: Cancer care as a forgotten humanitarian priority

In humanitarian emergencies, the health agendas of international organizations and national response teams have historically prioritized acute and communicable diseases, trauma care, maternal-child health, and malnutrition. These priorities are understandable given the immediate threats to life in displaced and crisis-affected populations. However, this acute focus has inadvertently marginalized the long-term, resource-intensive needs of patients suffering from non-communicable diseases (NCDs), especially cancer, which require complex diagnostic pathways, sustained treatment regimens, and palliative care infrastructure [4, 13].

Global guidelines and operational frameworks used during humanitarian responses have largely failed to integrate oncology into core planning. The Sphere Handbook, one of the most influential documents setting minimum standards for humanitarian response, does not include specific provisions for cancer care, despite acknowledging the growing burden of NCDs [12]. Similarly, the Inter-Agency Standing Committee (IASC) guidelines, WHO’s Emergency Medical Team (EMT) classifications, and emergency health cluster protocols offer no systematic guidance on the continuity of oncology services during crises [14, 15]. As a result, displaced cancer patients often experience delayed diagnoses, treatment discontinuation, and a lack of pain management, leading to preventable suffering and premature death.

This exclusion reflects a broader structural neglect of cancer care in global health governance, particularly in settings characterized by instability, weak health systems, and mass displacement. For decades, the architecture of global health - dominated by vertical programs targeting diseases such as HIV/AIDS, tuberculosis, and malaria - has sidelined NCDs in both funding and operational scope [16, 17]. Major global financing instruments, including the Global Fund and Gavi, do not cover cancer, and NCDs receive less than 2% of humanitarian health funding globally, despite accounting for the majority of disease burden in many conflict zones [18, 19].

In addition, humanitarian organizations face practical and ethical challenges in delivering cancer care: limited access to radiotherapy and chemotherapy, inadequate pathology services, and lack of specialists in the field. Many NGOs are reluctant to initiate cancer treatment in refugee camps or field hospitals due to the long-term nature of care, the fear of treatment interruption, and the absence of referral pathways to tertiary care centers [9, 20]. Even when oncology services are available, patients must often navigate unclear legal entitlements, financial hardship, and social stigma, particularly those living in host countries where refugees lack healthcare coverage or legal residency [21, 22].

The invisibility of oncology in humanitarian settings is not simply a gap in service delivery - it is a policy failure. The absence of normative guidance, financing mechanisms, and cross-sectoral strategies has allowed oncology needs to remain peripheral in crisis response, despite their growing relevance. As conflicts become protracted and displacement becomes semi-permanent, the failure to integrate cancer care into humanitarian policy frameworks raises urgent questions of global health equity, justice, and accountability.

A reconfiguration of humanitarian health priorities is essential - one that acknowledges cancer not as a secondary concern but as a critical component of dignified, comprehensive care for displaced and vulnerable populations. This shift demands inclusion of oncology in global humanitarian standards, creation of NCD-sensitive funding models, and development of scalable, context-specific oncology protocols suited for crisis environments.

### Cancer in crisis: Epidemiology and access challenges among migrants and refugees

The global refugee population exceeded 117 million in 2024, marking the highest number of forcibly displaced persons in recorded history [23]. While acute medical concerns such as injuries, infectious diseases, and malnutrition dominate emergency response agendas, the rising burden of non-communicable diseases (NCDs) - especially cancer - among migrants and refugees has received insufficient attention [2, 7]. Cancer is increasingly recognized as a critical but underreported cause of morbidity and mortality in humanitarian contexts, particularly in protracted crises and densely populated refugee settlements.

Recent studies indicate that cancer incidence in refugee populations often mirrors or even exceeds host country rates due to delayed diagnoses, exposure to carcinogens (e.g., in conflict zones or refugee camps), and poor living conditions [22]. For instance, in Jordan - home to large Syrian and Iraqi refugee populations - oncology units reported significant numbers of advanced-stage breast, colorectal, and hematologic malignancies among displaced persons, often diagnosed too late for curative treatment [13]. Similarly, reports from Lebanon’s Bekaa Valley and Turkey’s southern provinces underscore the growing cancer caseloads in regions with limited oncologic infrastructure and inconsistent donor support [21, 24].

Access to cancer care for displaced people is impeded by a constellation of systemic, legal, and logistical barriers. One of the primary challenges is the legal status of refugees, asylum seekers, and undocumented migrants, which affects their eligibility for public health services in host countries. In many cases, lack of legal documentation excludes these populations from national health insurance schemes, specialist referrals, or subsidized medications [3, 25]. Even where entitlement exists, bureaucratic delays and language barriers further restrict access. The absence of comprehensive cancer registries or epidemiological surveillance systems in refugee camps and transit zones also limits the ability to design targeted interventions, track outcomes, or anticipate treatment needs [18, 26].

Disrupted diagnostic pathways - including the unavailability of imaging, histopathology, and genetic testing - pose another critical challenge. In humanitarian settings, healthcare infrastructure is often fragile, lacking capacity for even basic screening programs for cancers such as cervical, breast, or colorectal [17]. Chemotherapy, radiotherapy, and surgical services are rarely available on-site and frequently require referrals to urban tertiary hospitals, which may be politically or geographically inaccessible to displaced patients [23].

Certain population subgroups experience compounded vulnerability. Women, for example, face stigma and social isolation that discourage seeking care for cancers such as breast or cervical cancer. In many traditional refugee communities, discussing gynecological symptoms or undergoing pelvic exams is taboo, delaying diagnoses and treatment [27]. Additionally, gender-based violence in displacement contexts increases risks for cervical cancer through higher rates of HPV transmission [28]. Children with cancer in humanitarian settings often face near-total exclusion from care due to the resource-intensive nature of pediatric oncology and the lack of specialized personnel or infrastructure [29]. Elderly refugees, who often have multiple comorbidities and reduced mobility, are less likely to be prioritized for treatment and may be excluded from host-country health entitlements altogether [30].

Inadequate pain relief and palliative care further exacerbate suffering. Opioid access is severely restricted in many crisis zones due to security, regulatory, and supply chain issues, leaving terminal cancer patients with little or no analgesia [31]. This situation not only violates international palliative care norms but also highlights the moral failure of existing systems to ensure dignity for displaced individuals with terminal illness.

Overall, the epidemiological reality of cancer in refugee and migrant populations remains poorly understood, under-documented, and systematically neglected. Addressing these gaps requires investment in mobile cancer screening programs, cross-border referral mechanisms, legally inclusive health policies, and robust epidemiological surveillance - especially in regions experiencing chronic or cyclical displacement.

### Case studies from conflict zones

Conflict zones represent some of the most hostile environments for the provision of cancer care. In such contexts, the disruption of infrastructure, displacement of health professionals, blockade of essential supplies, and collapse of public health institutions contribute to significant delays in diagnosis and discontinuities in cancer treatment. Three contemporary humanitarian emergencies - Gaza, Sudan, and Ukraine - offer critical case studies on how cancer care is neglected or adapted in war-affected populations. These cases illustrate both the failures of existing humanitarian responses and emerging opportunities for resilient, transnational oncology frameworks.

#### Gaza: Cancer care under siege

The Gaza Strip, under blockade since 2007, represents a chronic humanitarian emergency where access to cancer care is severely restricted by political and logistical constraints. Gaza has one public oncology unit at the Turkish-Palestinian Friendship Hospital, but chemotherapy is inconsistently available, and radiotherapy is entirely absent due to Israel’s prohibition on importing radiological equipment [9].

A 2022 study found that over 60% of cancer patients in Gaza required referral to hospitals outside the enclave, primarily in East Jerusalem or the West Bank, yet only about half of these referrals were approved on time by Israeli authorities [32]. Pediatric cancer patients face particularly poor outcomes due to treatment delays and absence of specialized care. Delays in permit approvals range from days to several weeks, during which time patients miss treatment windows for surgery or chemotherapy [33].

Humanitarian organizations such as WHO and Médecins Sans Frontières (MSF) have attempted to mediate access, but efforts remain fragmented and largely reactive. Moreover, political instability has undermined the development of local oncology capacity, and cancer patients have been disproportionately impacted by recent escalations, with health facilities being targeted or damaged during airstrikes [34].

#### Sudan: Health system collapse and oncology neglect

The outbreak of armed conflict in Sudan in April 2023 between rival military factions has pushed the country into a full-blown health crisis. The National Cancer Institute in Wad Madani, one of the few functioning oncology centers, was forced to shut down, and healthcare workers have been displaced or killed [35]. Khartoum’s oncology facilities have been looted, leaving cancer patients without any access to diagnostic or treatment services.

Sudan’s pre-conflict oncology infrastructure was already limited - with only 3 radiotherapy machines for a population of 45 million - but the war has reduced access to near zero in most regions [36]. Internally displaced patients have flooded peripheral areas such as Port Sudan, where services are overstretched and primarily oriented toward trauma and infectious disease care. International response efforts have focused primarily on acute malnutrition, cholera, and maternal health, with cancer care virtually omitted from emergency health packages [37].

The WHO has identified oncology supply chain restoration as a medium-term priority, but no formal strategy exists for reinstating cancer services in conflict-affected zones. Refugees fleeing Sudan into Chad and Egypt also face barriers to treatment due to lack of integration into national health systems and unclear referral mechanisms [38].

#### Ukraine: managing continuity of cancer care during wartime displacement

Unlike Gaza and Sudan, Ukraine provides a contrasting case in which existing health infrastructure was relatively strong prior to conflict, and cross-border coordination with European Union (EU) nations helped facilitate care continuity. Following Russia’s full-scale invasion in 2022, millions of Ukrainians were displaced internally and across borders - many of whom were cancer patients on active treatment [39].

The Ukrainian Ministry of Health and the European Cancer Organisation coordinated early to establish a cross-border cancer care registry and referral network, allowing patients to resume chemotherapy or radiotherapy in host countries such as Poland, Germany, and Romania [40]. EU countries rapidly adjusted health policies to accept Ukrainian patients without delay, often granting them temporary health entitlements, fast-tracked diagnostics, and even access to clinical trials [41].

Despite these successes, challenges remain. Many Ukrainian oncology centers were destroyed or damaged in Russian attacks - notably in Kharkiv, Mariupol, and Severodonetsk - and displaced elderly patients or those in rural areas struggle to access care even within Ukraine [42]. Mental health comorbidities, high transport costs, and linguistic barriers have complicated care for refugees, and some patients face disruptions when transitioning between national health systems.

#### Comparative insights: Challenges and response gaps

To synthesize the policy and access implications of these case studies, the Table 1 outlines the comparative status of oncology care in the three conflict settings:

**Table 1:** Comparative analysis of oncology care in three conflict zones

Country	Key challenges	Response mechanisms	Policy gaps
Gaza	No radiotherapy; surgical referrals delayed; political blockade	NGO advocacy, cross-border permit system	Lack of sovereignty over health system; political interference in care access
Sudan	Total collapse of oncology infrastructure; personnel displacement	Minimal; emergency focus on infectious diseases	Oncology excluded from emergency response and health cluster priorities
Ukraine	Destruction of facilities; displaced patients across EU	EU-wide registry, fast-tracked cross-border care	Fragmented access for rural/elderly; limited psychosocial support

These case studies highlight the urgent need for a harmonized, rights-based global policy framework that ensures oncology services are embedded into humanitarian response protocols. The effectiveness of Ukraine’s regional response model illustrates the potential of international cooperation, while the failures in Gaza and Sudan emphasize how political and infrastructural barriers can render cancer care inaccessible in the absence of proactive systems.

### Ethical dilemmas in oncology triage in crisis settings

The delivery of cancer care in humanitarian and conflict-affected settings presents complex ethical challenges. Oncology, as a resource-intensive specialty, often demands high-cost diagnostics, long-term treatments, and specialized infrastructure - resources that are scarce or altogether absent in crisis contexts. When faced with such scarcity, decision-makers must grapple with triage decisions that inherently raise ethical questions of justice, prioritization, and the balance between curative and palliative intentions. These dilemmas intersect with broader humanitarian principles and challenge conventional frameworks of global health equity.

#### Scarcity, prioritization, and the rationing of cancer care

In humanitarian crises, oncology services are frequently deprioritized in favor of acute emergency responses such as trauma care, infectious disease containment, or maternal health. This deprioritization is often justified by the perception that cancer care is non-urgent, resource-heavy, and less cost-effective in low-resource emergency settings [2]. As a result, health providers must ration care - deciding which patients receive chemotherapy, who gets diagnostic scans, or whether to initiate radiotherapy that cannot be sustained due to displacement risks.

Triage protocols in oncology during crises are often improvised or non-existent, forcing frontline clinicians and humanitarian actors to make ad hoc decisions without ethical guidance or policy frameworks [43]. In practice, this has led to implicit exclusion criteria based on prognosis, mobility, age, or perceived “treatability,” which may conflict with patient-centered ethical norms and rights-based health care models [44].

#### Balancing curative and palliative care in humanitarian crises

One of the most profound ethical tensions is the allocation of limited resources between curative intent interventions and palliative or supportive care. In conflict zones like Sudan or Yemen, where health infrastructure is decimated, it may be ethically and logistically infeasible to offer curative therapies for advanced cancers. In such contexts, emphasis shifts toward pain management, psychosocial support, and dignified end-of-life care [45].

However, the provision of palliative care is often overlooked in humanitarian settings, even though it is more feasible than curative treatment. Studies indicate that less than 5% of humanitarian medical missions include formal palliative care services, despite high demand for such support among cancer patients and their families [46]. WHO has called for integrating palliative care into emergency medical packages, yet implementation remains sparse due to lack of training, morphine availability, and cultural stigmas [47].

The ethical dilemma arises in choosing whether to invest in life-prolonging treatments for a few, or symptom relief and dignity for many. The principle of proportionality - doing the greatest good with the least harm - often guides such decisions, but must be balanced against the duty to care and respect for individual patient dignity [48].

#### Justice, equity, and cross-national responsibility

Ethical oncology triage during crises cannot be disentangled from global justice frameworks. Refugees and migrants often fall into legal and moral grey zones, excluded from national cancer programs due to lack of documentation, citizenship, or financial means [49]. The ethical principle of distributive justice requires that access to life-saving or life-extending cancer treatments not be determined solely by geography or political status.

The cross-border nature of many conflicts (e.g., Ukraine to Poland, Sudan to Egypt, Syria to Lebanon) raises additional dilemmas of shared responsibility. Host nations may be overwhelmed, under-resourced, or unwilling to extend cancer services to non-citizens, despite international legal obligations under the International Covenant on Economic, Social and Cultural Rights [50]. Moreover, global donors often prioritize short-term emergency care over chronic disease management, reflecting an ethically problematic hierarchy of needs [51].

International oncology societies and ethical boards have called for the creation of transnational triage frameworks - ethical guidelines and logistical pathways to prioritize patients across borders based on urgency, benefit potential, and equity [52]. These would require coordination between humanitarian actors, national health ministries, and international funders, including mechanisms for ethical review and appeals in triage decisions. Ethical Dilemmas and Decision Points in Crisis Oncology are depicted in Table 2.

**Table 2:** Ethical dilemmas and decision points in crisis oncology

Ethical domain	Core dilemma	Contextual examples	Suggested ethical principle
Resource Allocation	Who gets chemotherapy, surgery, or radiation in scarcity?	Gaza (permit delays), Sudan (system collapse)	Distributive justice, Triage fairness
Curative vs. Palliative	Should care focus on cure or comfort in late-stage cancers?	Yemen, Eastern DRC	Proportionality, Patient dignity
Access for Non-citizens	Are refugees/migrants entitled to full oncology care?	Ukraine refugees in EU, Syrian refugees in Lebanon	Equity, Right to health
Cross-border Coordination	Who is responsible for financing and delivering care?	Sudanese patients in Chad; Gazans needing referrals to Israel	Global solidarity, Shared responsibility

These dilemmas underscore the need to move beyond charity-based humanitarianism and toward rights-based, ethically accountable cancer care models. Ethical frameworks in oncology must evolve to accommodate crisis realities without abandoning core values of autonomy, equity, and justice. Triage systems that are transparent, participatory, and aligned with international human rights law are essential for ethical oncology in emergency settings.

### Towards cross-border continuity of oncology care

As protracted conflicts, forced displacement, and fragile health systems increasingly interrupt access to cancer diagnosis and treatment, there is an urgent need to rethink oncology care as a cross-border health responsibility. Unlike infectious disease outbreaks that prompt immediate international response, non-communicable diseases like cancer often lack operational frameworks for continuity of care across national boundaries [53]. In response to this gap, emerging policy models advocate for regional, bilateral, or multilateral approaches to guarantee care pathways for displaced cancer patients, supported by international legal, humanitarian, and medical coordination.

#### Bilateral and multilateral cross-border treatment agreements

One pragmatic solution to disrupted oncology access is the formalization of bilateral or multilateral treatment agreements between neighboring countries. Such arrangements can allow refugee or conflict-affected populations to receive diagnostic or therapeutic services in a nearby stable country with stronger oncology infrastructure. This has precedent: during the Syrian conflict, Lebanon and Jordan established referral mechanisms to tertiary cancer centers for displaced Syrians, although inconsistently and with heavy reliance on donor support [54].

More structured and equitable frameworks are needed, ideally codified through memoranda of understanding (MoUs) that include cost-sharing, documentation protocols, and guarantees of follow-up care. For example, Poland, Slovakia, and Hungary have collaborated to provide cancer treatment to Ukrainian refugees displaced during the 2022 Russian invasion, with support from EU emergency funds and UNHCR coordination [55]. These efforts highlight the feasibility of cross-border cancer care when political will, regional cooperation, and donor engagement align.

Such agreements must also account for longitudinal care needs, including maintenance chemotherapy, radiotherapy regimens, and post-operative monitoring. This necessitates not just episodic access, but an integrated continuum of care that may extend months or years beyond initial displacement [56].

#### Leveraging humanitarian corridors for oncology access

Humanitarian corridors - secure passageways negotiated to deliver aid or evacuate civilians - can also serve as vital conduits for transporting cancer patients to treatment centers. These corridors have been used in Gaza, Ukraine, and parts of Sudan to evacuate critical patients needing dialysis, trauma surgery, or chemotherapy, albeit sporadically and often with political delays [57].

Institutionalizing oncology access within these humanitarian logistics corridors can expand their function beyond acute trauma to include chronic and life-threatening diseases. For example, the World Health Organization (WHO), working with the Egyptian and Palestinian Red Crescent societies, has coordinated patient transport from Gaza to hospitals in Egypt and Israel for cancer care, though these transfers are vulnerable to security dynamics and bureaucratic delays [58].

To improve predictability and equity, oncology-specific corridors could be pre-negotiated during ceasefires or in parallel to broader humanitarian operations, with clear triage criteria, referral processes, and follow-up systems embedded in the corridor protocol. Technology-enabled patient tracking and digital health records can facilitate continuity despite cross-border transitions.

#### Role of international NGOs and WHO in coordinating care

International non-governmental organizations (NGOs), along with intergovernmental agencies such as the World Health Organization (WHO), play a central role in operationalizing and scaling cross-border oncology care. Organizations like Médecins Sans Frontières (MSF), the International Organization for Migration (IOM), and the International Cancer Control Partnership (ICCP) have increasingly incorporated cancer care in humanitarian programming, especially for palliative and pediatric oncology [59].

These actors can provide bridging services, including funding for referrals, mobile oncology units, and cancer medication logistics. WHO’s Emergency Medical Teams (EMT) initiative has also begun including non-communicable disease specialists in humanitarian deployments, although systematic integration of oncology remains limited [60].

An essential gap remains the lack of centralized global coordination for displaced cancer patients, including registries, referral platforms, and financing mechanisms. The WHO could spearhead an International Oncology Continuity Taskforce to coordinate treatment among national systems, pool funding (e.g., through the WHO Contingency Fund for Emergencies), and standardize triage and referral protocols based on clinical urgency and vulnerability [61].

In addition, the use of digital health tools - such as interoperable electronic health records (EHRs), tele-oncology platforms, and blockchain-secured patient IDs - can help ensure that displaced patients do not fall through the cracks as they move between systems and jurisdictions [62]. Proposed Models for Cross-Border Oncology Continuity are depicted in Table 3.

**Table 3:** Proposed models for cross-border oncology continuity

Mechanism	Key Features	Examples	Challenges	Facilitating actors
Bilateral/Multilateral Agreements	MoUs between countries; cost-sharing; treatment referrals	Ukraine–Poland–EU, Syria–Jordan	Political will, sustainability	National health ministries, EU, UNHCR
Humanitarian Corridors	Safe passage for urgent cancer care; logistical protocols	Gaza–Egypt, Ukraine–EU	Security risks, bureaucracy	WHO, Red Crescent, MSF
NGO-led Coordination	Mobile units, palliative care, diagnostics	MSF, ICCP, IOM projects	Fragmented efforts, funding gaps	NGOs, donors, philanthropic consortia
WHO-led Global Platform	Cross-national oncology registry; treatment matching	(Proposed) Oncology Continuity Taskforce	Global buy-in, interoperability	WHO, GAVI, World Bank

The path forward must transition from reactive, case-by-case cancer care for displaced people to a systematized, rights-based, cross-border oncology framework. Political agreements, humanitarian logistics, digital innovations, and global coordination mechanisms together can embed cancer care as a protected, portable service - regardless of national origin or displacement status.

### Tele-oncology and mobile units: Technology-enabled solutions

In conflict-affected and humanitarian contexts where access to specialized cancer services is severely limited, tele-oncology and mobile healthcare units have emerged as innovative, adaptive solutions to bridge critical gaps in oncology care. These modalities offer not only flexibility and rapid deployment, but also enable decentralized, equitable access to expert oncology services without the need for full-fledged cancer centers, which are often unfeasible in war zones or refugee camps.

#### Successful models in crisis settings

A number of field-tested initiatives illustrate the feasibility and impact of tele-oncology in crisis and remote settings:

- Médecins Sans Frontières (MSF) has piloted mobile oncology clinics and telemedicine consultations in various humanitarian zones, including Yemen and the Central African Republic, using digital imaging and remote oncologist networks to assist with diagnosis and therapy planning [63].
- Project ECHO (Extension for Community Healthcare Outcomes), originally launched in New Mexico, has been successfully adapted to support oncology care in underserved regions by connecting local providers with multidisciplinary expert teams through virtual tumor boards. Its oncology hubs now extend to East Africa and conflict-affected regions in the Middle East [64, 65].
- The Jordan Health Aid Society, in partnership with the WHO and local mobile health platforms, has provided breast cancer screening and follow-up for Syrian refugee women using digital mammography and remote reading protocols [66].

Such models demonstrate that when supported by modest infrastructure and capacity building, tele-oncology can deliver diagnostics, decision support, and even training in real-time - even under duress.

#### Opportunities for diagnostics, second opinions, and treatment planning

Tele-oncology enables remote pathology consultation, radiologic image interpretation, and treatment planning across borders, especially when clinical oncology expertise is sparse in the host country. For example, second-opinion services facilitated by networks like the International Cancer Expert Corps (ICEC) or ASCO’s Global Oncology programs allow local providers in refugee zones to optimize protocols based on current evidence and individualized patient needs [67, 68].

Mobile units, equipped with portable ultrasound, mammography, and laboratory testing platforms, can serve as point-of-care diagnostic hubs in camps and isolated border settlements [69]. These mobile centers can also act as collection points for biospecimens - such as fine-needle aspirations or biopsy slides - that are digitally transmitted to centralized pathology labs via telepathology systems [70].

Furthermore, treatment planning support, including chemotherapy protocol design, toxicity monitoring, and palliative care approaches, can be facilitated virtually. Real-time video consultations between host-country physicians and international oncologists can significantly improve outcomes and patient confidence, particularly in complex pediatric or hematologic malignancies [71].

#### Implementation challenges

Despite the promise of these technologies, there are notable barriers to implementation:

- Digital Infrastructure: Many refugee settings lack stable internet, reliable electricity, and the hardware needed for imaging transmission or video consultations. Satellite internet or solar-powered solutions have been piloted but remain costly [72].
- Data Privacy and Regulation: Jurisdictional ambiguity around medical licensing, cross-border consultation legality, and patient data sovereignty hinders scaling of tele-oncology, particularly when systems lack GDPR-equivalent protections [73].
- Language and Cultural Barriers: Remote communication often breaks down due to lack of interpreters, misaligned cultural understandings of cancer, or divergent expectations between local providers and remote specialists [74].
- Sustainability: Many tele-oncology initiatives are donor-funded or pilot-based, lacking sustained integration into national health systems or refugee response plans [75].

Summary of Tele-Oncology and Mobile Unit Innovations in Crisis Settings is depicted in Table 4.

**Table 4:** Summary of tele-oncology and mobile unit innovations in crisis settings

Model	Functions	Geography	Strengths	Challenges
MSF Mobile Oncology Units	Diagnostics, teleconsultation, palliative care	Yemen, CAR	Rapid deployment, adaptability	Cost, follow-up limitations
Project ECHO Oncology Hubs	Second opinions, tumor boards, capacity building	East Africa, Middle East	Scalable education, specialist access	Connectivity, time zones
Jordan Breast Screening via Tele-Mammography	Remote imaging and follow-up	Refugee camps, Jordan	Women’s cancer focus, early detection	Screening continuity
ICEC Tele-Consultations	Remote pathology, protocol optimization	Global	High-quality second opinions	Licensing and data sharing constraints

### Legal and policy recommendations for equitable migrant oncology care

The recognition of cancer as a humanitarian priority requires a paradigm shift in how global health policies address the intersection of displacement, non-communicable diseases (NCDs), and access to specialized care. Current humanitarian responses are largely designed around acute infectious diseases, trauma, and maternal-child health, resulting in a systemic policy void for oncology services among migrants, asylum seekers, and refugees. This section outlines key legal and policy strategies to rectify this neglect and promote sustainable, equitable access to cancer care for displaced populations.

#### Harmonizing refugee health rights with oncology access

The 1951 Refugee Convention and its 1967 Protocol provide the legal basis for the right to health among refugees; however, these instruments do not explicitly define the scope of health services, and implementation varies across host nations [76]. Cancer care is often excluded or deprioritized under the presumption of high costs and limited resources. Yet the International Covenant on Economic, Social and Cultural Rights (ICESCR) affirms the right to the highest attainable standard of physical and mental health, which includes treatment for chronic and life-threatening illnesses like cancer [77].

There is an urgent need to harmonize these rights with practical entitlements by integrating oncology into national refugee health policies. Countries such as Jordan and Turkey have made notable advances by including selected cancer services in refugee health benefit packages [78, 79]. However, these are often donor-dependent, underscoring the need for robust legal frameworks that ensure non-discriminatory access to oncology irrespective of legal status.

Statutory alignment between health ministries and immigration authorities, as seen in the EU’s Temporary Protection Directive (TPD) during the Ukrainian refugee crisis, can ensure that displaced populations are enrolled into host nation oncology pathways without bureaucratic delay [80].

#### Funding models for cross-border cancer care

The high cost of cancer diagnostics and treatment remains a central barrier to equity. Effective financing mechanisms must combine domestic allocations, international aid, and innovative financing instruments. Three promising approaches include:

- Pooled International Risk-Sharing Funds: Modeled after the Global Fund for AIDS, TB, and Malaria, a Cancer in Crisis Fund could allocate resources based on population displacement and burden of disease. Advocacy for this is growing, particularly under the NCD Alliance and UICC [81].
- Public–Private Partnerships (PPPs): Collaborations with pharmaceutical companies, like the GAVI–Roche HER2 therapy initiative for refugees in Lebanon, show promise in delivering high-cost therapies at subsidized rates [82].
- Bilateral Reimbursement Agreements: Countries receiving displaced populations (e.g., Poland and Germany post-Ukraine conflict) have explored reciprocal agreements to reimburse care across borders, particularly for high-need cases like pediatric or metastatic cancers [83].

The integration of cancer care in UNHCR’s Global Compact on Refugees operational programming is also a critical step, enabling refugee-hosting countries to access shared global responsibility and funding mechanisms [84].

#### Integrating NCDs into humanitarian response frameworks

Despite the World Health Organization’s (WHO) increasing acknowledgment of NCDs in emergencies, oncology care is still largely absent from standard humanitarian health protocols such as the Sphere Standards or the Inter-Agency Standing Committee (IASC) guidelines [12]. A critical revision of these frameworks is needed to include:

- Cancer-specific service packages under the WHO Emergency Medical Teams (EMT) classification, including diagnostics, chemotherapy, palliative care, and psychosocial support [58].
- Rapid cancer needs assessments as part of humanitarian health cluster evaluations, with disaggregated data collection for age, gender, and cancer type.
- Dedicated oncology focal points within the WHO Health Emergency Programme and UNHCR’s Public Health Section to coordinate technical guidance and partner collaboration.

Table 5 summarizes key legal and policy recommendations for integrating oncology into migrant and refugee health systems.

**Table 5:** Legal and policy levers to strengthen migrant oncology care

Policy area	Recommendation	Current gaps	Best practice examples
Health Rights Alignment	Incorporate oncology into national refugee health policies	Vague entitlements, exclusion of chronic care	Jordan’s Cancer Access Protocol (2019)
Funding Models	Establish global pooled funds and PPPs for displaced cancer patients	High cost, donor dependency	Lebanon HER2 therapy pilot (GAVI–Roche)
Legal Harmonization	Use directives like EU’s TPD for cancer enrollment in host systems	Fragmented access, legal delays	Poland’s Ukrainian cancer care model (2022)
Humanitarian Frameworks	Revise Sphere and EMT standards to include NCDs	Acute care-centric guidelines	WHO EMT Type 2+ NCD expansion pilot (Philippines, 2023)
Cross-border Continuity	Bilateral treatment and reimbursement agreements	Lack of portability in care plans	Germany–Ukraine Oncology MOU (2022)

### Research gaps and data deficits

Effective cancer policy planning and service provision for migrant, refugee, and conflict-affected populations are significantly hindered by severe gaps in data availability, quality, and accessibility. While the burden of cancer among forcibly displaced populations is rising, the evidence base to inform needs-based, equitable oncology responses remains dangerously thin. This section outlines the current deficiencies in epidemiological surveillance, registries, ethical research mechanisms, and data-sharing protocols, underscoring their implications for clinical outcomes and policy design (Table 6).

**Table 6:** Key research and data gaps in migrant oncology care

Domain	Current gap	Implications	Suggested action
Epidemiology	No disaggregated data by migrant status	Invisible burden, policy blind spots	Integrate migration status into all cancer surveillance systems
Registries	Absent in refugee-hosting or conflict-affected areas	Lack of continuity of care, no outcome tracking	Develop mobile, interoperable registries with real-time syncing
Ethical Research	No adapted protocols for oncology research in crises	Exploitation risk, poor consent quality	Implement crisis-specific IRB standards and data protection norms
Data Sharing	Limited interoperability across agencies or borders	Duplicative efforts, fragmented care	Establish regional cross-border oncology data hubs
Gender and Age Disaggregation	Minimal in conflict settings	Missed vulnerabilities in women and elderly	Include age-sex breakdown in all data fields by default

#### Absence of disaggregated data on migrant cancer care

Most global and national cancer surveillance systems do not capture migration status as a routine demographic variable, resulting in an invisible epidemiology of cancer in displaced and mobile populations [85]. Data is often subsumed under general categories like “foreign-born” or “non-citizens,” which fails to differentiate between refugees, asylum seekers, internally displaced persons (IDPs), and undocumented migrants [86]. This lack of granularity conceals variations in exposure risk, health-seeking behavior, screening uptake, and survival outcomes.

Moreover, conflict-affected zones and refugee-hosting regions often experience health system collapse, where data collection becomes logistically unfeasible. For example, Syria’s national cancer registry ceased to function effectively after 2011, and data from Gaza or South Sudan is virtually nonexistent [87, 88].

#### Need for registries, surveillance, and shared databases

Developing context-specific, ethically governed cancer registries in humanitarian and fragile settings is crucial to closing the evidence gap. Registries should collect disaggregated data by age, sex, cancer type, legal status, and displacement history. Several promising efforts exist:

- The Global Initiative for Cancer Registry Development (GICR), led by IARC and WHO, supports low-resource countries in establishing population-based cancer registries [89]. However, their reach into humanitarian contexts is limited.
- The Surveillance, Epidemiology, and End Results (SEER) Program in the U.S. has piloted immigrant-status-linked oncology datasets, offering a potential model for replication in high-income refugee-hosting states [90].
- The EMPHNET Cancer Surveillance Project in the MENA region is exploring regional harmonization of cancer data across conflict-affected countries, though it remains underfunded [91].

To be effective, these registries must be interoperable across borders, while respecting data protection laws such as GDPR in Europe and host-country legislation. They should also enable real-time data sharing during mass displacement crises to allow care continuity tracking, treatment matching, and outcome evaluation.

#### Ethical research frameworks in conflict zones

Conducting cancer research in humanitarian settings involves unique ethical and operational complexities. These include obtaining informed consent in low-literacy or high-trauma environments, ensuring participant safety, avoiding therapeutic misconception, and protecting privacy during displacement [92].

The Declaration of Helsinki, while providing broad ethical guidance, lacks detailed operational provisions for conflict zones. More context-specific instruments are needed - such as those proposed in the REMAP project (Research Ethics in Mass Population Displacement), which advocates for situationally adaptive consent models and rapid ethics review mechanisms [93].

Institutional review boards (IRBs) and humanitarian research ethics committees should prioritize research that:

- Avoids duplicative data extraction from vulnerable groups;
- Shares benefits with host communities;
- Employs participatory methods that center the voices of displaced cancer patients [94].

Moreover, all data collected must serve a translational purpose, directly informing service improvement, policy revisions, or funding realignment. Research must not be extractive, and participants should retain agency and feedback rights regarding outcomes.

## DISCUSSION

The findings of this review underscore the acute marginalization of oncology within humanitarian health policy and practice frameworks. While infectious diseases, maternal health, and trauma care dominate global emergency responses, non-communicable diseases (NCDs) - and particularly cancer - have been persistently overlooked in both funding priorities and operational planning in conflict and displacement contexts [69, 70]. This omission occurs despite growing epidemiological evidence indicating a rising cancer burden among refugees, asylum seekers, and internally displaced persons (IDPs), particularly in protracted crises where populations remain displaced for years or decades [95, 96].

The structural neglect of cancer care in humanitarian settings is partially rooted in the historical dichotomy between acute emergency responses and chronic disease management. Most humanitarian actors have prioritized interventions that deliver immediate, visible outcomes, often under the constraints of limited time and resources [97]. However, this model is increasingly misaligned with the realities of modern displacement, where long-term health needs - including cancer screening, treatment, and palliative care - are urgent and growing. The absence of oncology-specific standards in humanitarian frameworks such as the Sphere Handbook or WHO Emergency Medical Teams (EMT) typologies illustrates this disconnect [12, 98].

Case studies from conflict-affected zones - such as Gaza, Sudan, and Ukraine - illustrate the devastating impact of disrupted oncology services on displaced populations. In Gaza, for example, over 60% of cancer patients lack timely access to radiotherapy due to border closures and dependency on external referrals, resulting in increased mortality and treatment abandonment [99]. Sudan’s health system collapse following armed conflict has virtually dismantled oncology infrastructure, while the Ukrainian war has displaced tens of thousands of cancer patients across borders into EU countries, necessitating transnational coordination for continuity of care [100–102]. These cases highlight not only the clinical consequences of disrupted care, but also the policy vacuum at both national and international levels.

In response, this review proposes a rethinking of oncology care delivery that includes cross-border treatment agreements, activation of humanitarian corridors, and the integration of cancer services into emergency planning through platforms like the WHO EMT or the Inter-Agency Standing Committee (IASC) [103]. Tele-oncology and mobile cancer units also emerge as critical technological enablers that can bridge geographical and logistical gaps. Programs such as Project ECHO and MSF’s mobile palliative care services demonstrate the feasibility of deploying digital and mobile platforms for cancer diagnosis, second opinions, and even chemotherapy support in resource-constrained settings [104, 105]. Nevertheless, these models require significant investment in digital infrastructure, human resource capacity, and legal harmonization across jurisdictions.

Ethical dilemmas in oncology triage further complicate care delivery in crisis contexts. Decisions about prioritization, especially when resources are scarce, necessitate frameworks grounded in distributive justice, medical utility, and the rights of displaced persons. The tension between curative and palliative goals must be negotiated transparently, with consideration for local cultural contexts, prognostic limitations, and the evolving nature of conflicts [106]. Given the political sensitivity surrounding refugee health entitlements, ethical frameworks should also be linked to policy instruments that ensure non-discrimination and uphold the principle of health as a human right [107].

The research gaps identified - particularly the absence of disaggregated data, functional registries, and interoperable cancer surveillance systems - are not merely technical deficits but reflect systemic policy inattention to migrant oncology care. Without robust data, displaced populations remain invisible in cancer control strategies, and health systems are unable to plan resource allocation or evaluate outcomes effectively [108]. Ethical research frameworks, such as those proposed by the REMAP project, offer guidance on conducting context-sensitive oncology research in humanitarian settings, including adaptive consent mechanisms and community engagement protocols [109–111].

This discussion ultimately points to a necessary shift in humanitarian paradigms - from reactive, short-term medical relief to inclusive, rights-based health system strengthening that accounts for NCDs. Oncology must be mainstreamed into humanitarian health policies, funding mechanisms, and international response protocols. National governments, especially refugee-hosting states, have a duty to harmonize refugee health entitlements with national cancer programs, while international bodies such as UNHCR, WHO, and IOM must play a proactive coordinating role.

## CONCLUSIONS: A HUMANITARIAN IMPERATIVE FOR GLOBAL ONCOLOGY JUSTICE

The rising cancer burden among forcibly displaced populations - refugees, migrants, and those in conflict-affected zones - represents a growing humanitarian crisis that global oncology can no longer afford to overlook. This review has illuminated the stark policy vacuum surrounding cancer care in humanitarian contexts, characterized by fragmented service delivery, interrupted treatment pathways, and the absence of coordinated cross-border care strategies. The cumulative impact is a systemic failure to uphold the right to health for some of the world’s most vulnerable people, in direct contradiction to international humanitarian principles and global health equity goals.

Cancer care in crisis settings is not merely a clinical challenge - it is a moral imperative and a test of global solidarity. The current neglect reflects deep structural inequities embedded in global health governance, where oncology is often seen as too complex, too resource-intensive, or too politically fraught to integrate into emergency response mechanisms. Yet as this review has demonstrated through epidemiological evidence, case studies, and emerging technological models, oncology care is both feasible and essential in humanitarian settings when supported by the right frameworks, policies, and political will.

We call upon national governments, international health agencies, humanitarian organizations, and the global oncology community to act decisively. This includes integrating non-communicable disease management - including cancer - into emergency medical standards; developing legally binding cross-border care agreements; investing in tele-oncology and mobile diagnostics; and embedding oncology into refugee health entitlements and funding models. Ethical triage protocols, equitable resource allocation, and inclusive research frameworks must guide all interventions.

Ultimately, achieving cancer equity in humanitarian and conflict settings requires reframing oncology not as a luxury of stable systems, but as an indispensable component of human dignity and global justice. The failure to act is not a matter of capacity alone, but of conscience.
